# Medical College and Hospital Notes

**Published:** 1898-09

**Authors:** 


### MEDICAL COLLEGE AND HOSPITAL NOTES.

When the movement looking to the establishment of a state laboratory for the investigation of cancer was inaugurated, the faculty of the University of Buffalo met with many difficulties and for a time the task seemed a hopeless one. Finally, just when things were darkest, Mr. E. H. Butler, proprietor of the *Buffalo Evening News*, became interested in the project and gave the faculty such assistance and support as to finally attain the object so earnestly desired. In recognition of his assistance the following resolution was passed and in handsomely engrossed form was presented to Mr. Butler:

University of Buffalo. The medical faculty desire to place on record their appreciation of the great service which Edward H. Butler has rendered them by his hearty interest and zealous coöperation in their endeavor to provide for the scientific study of cancer, and hereby extend to him their most sincere and hearty thanks, bespeaking at the same time his continued interest in their efforts.

For the Faculty,

MATTHEW D. MANN, *Dean*.

Julius Pohlman, *Registrar*.

---

The seventeenth annual announcement of the New York Post-Graduate Medical School and Hospital, University of the State of New York, for 1898-99 has just been issued. It shows that 523 practitioners of medicine have attended its courses during the past year. They came from the various states of the Union and the Dominion of Canada. There were ten physicians from foreign countries, two of these being from India and one from Japan. Only ninety-six were from the state of New York.

---

THE Atlanta Medical College and the Southern Medical College, also located at Atlanta, have united, thus forming one strong institution, in the place of two with divided forces. This action is in accordance with the spirit of progression and improvement in medical education, and is to be commended on every hand as tending to strengthen the American medical profession.

---

THE next course of lectures in the Medical Department of the University of Buffalo will begin September 12th, at 8 o'clock P. M. Dr. Alvin A. Hubbell will deliver the opening address.

